# Initiatives and strategies for development of nanotechnology in nations: a lesson for Africa and other least developed countries

**DOI:** 10.1186/1556-276X-9-133

**Published:** 2014-03-20

**Authors:** Ikechukwu C Ezema, Peter O Ogbobe, Augustine D Omah

**Affiliations:** 1 Department of Metallurgical & Materials Engineering, University of Nigeria, Nsukka 410001, Enugu, Nigeria; 2 Technology Incubation Centre Enugu, Federal Ministry of Science & Technology, Abuja 400001, Nigeria

**Keywords:** Nanotechnology, Nations, Initiatives and strategies, Funding, Collaborations

## Abstract

It is a known fact that the progress and development of different nations of the world is strongly connected with the type of materials under their use. This paper highlighted the development of nanotechnology in some selected countries of the world through a careful review of their road maps by way of public and private initiatives, funding/investment profile, human resources development, industrial potentials, and focus in order to draw inferences. The peculiar challenges and opportunities for some African nations and other least developed countries (LDC) were drawn for their economic and technological developments. This investigation was simply based on open access literatures. The review showed that although nanotechnology is new globally, most countries of the world have had growing public and private investments aimed at bringing about new materials and systems that can impact positively on their economy and ensure their global competitiveness and sustainability. The global scenario suggests the crucial role of cooperation in a multidisciplinary collaboration/partnership between government ministries, agencies, institutions, and private sector/donor agencies in order to pool enough resource capital required for activities in nanotechnology.

## Review

### Introduction

Transformation in the materials world has been the bane of technological advancement worldwide as such human existence from generation to generation has been characterized by different materials under their use. This divides accordingly including the Stone Age, Bronze Age, Iron Age, Steel Age, Semiconductor Age, Advanced Materials (ceramic, polymer, and metal matrix composites) and now Nanomaterials/Nanocomposites [1]. Dr Butt [1] reported that such advancement ‘between 1900–1950 - gave rise to the manufacturing of radio, television and other electronic devices using analog inputs, while semiconductor micro chips were introduced between 1951–2000 to produce more sophisticated television, radio and small size computers, and internets.’ Since 2000, nanowires and nanodevices have been in use for characterization of more robust products.

Today many novel materials with high strength, light weight, and greater chemical resistance have come into existence and are grouped under nanomaterials [2], nanotubes (carbon nanotube (CNT)) [3], nanowires (light emitting diode (LED)), nanocrystals, and nanocatalysts [4]. Dr Butt [1] also reported that typical nanotechnology applications in various areas include but not limited to the following:

Energy - as in solar panels, fuel cells, batteries

Defense - as in producing special materials

Medicine/health - as in anti-cancer drugs, implants, dental pastes, diagnostic sensors

Environment and agriculture - as in water purification, animal drugs, crop quality, nanocapsules for herbicides, pesticides, insecticides and insect repellants, anti-toxicants, and filter.

Again, nanotechnology is now adopted in manufacturing of aerospace parts as nanocomposites - to improve its light weight and high strength structures and its lighting systems - using LED, popularly called low-energy saving bulbs.

Sargent [5] reported that some of the unique properties of nanoscience materials such as small size and high surface area to volume ratio have given rise to concerns about their potential implication on health, safety, and environment, particularly as regards to carbon nanotubes (CNTs). The truth is that research on the health risk of nanotechnology is at its collation stage [6-8] waiting for inference to be drawn and above all is the fact that the risk level is highly dependent on the potential to accumulate a reasonable quantity at a time rather than just having a contact [9].

Perhaps it is this uncertainty regarding health issues of nanotechnology activities that deters many countries from starting their own nanotechnology initiatives, but such position is a negative one because nanotechnology has come and it is fast growing into every area of life, and the earlier the surrounding challenges are confronted by a nation, organization, or agency, the better for her.

Many advanced countries such as USA, China, UK, Germany, Japan and many others have since a decade ago initiated and developed a robust nanotechnology plan for their respective countries. Also, few developing countries that have a clear understanding of the trend have in the recent past launched their own nanotechnology program and are today at various advanced stages with much economic benefits. Unfortunately, most African nations and some other least developed countries (LDC) have only demonstrated interest to start without any practical approach to its implementation. This paper therefore reviewed and highlighted some of the advanced nation's initiatives and strategies with a view to draw inferences that can help African nations and other LDC to initiate their own nanotechnology programs for their economic and industrial advancements.

### Nanotechnology programs of nations - initiatives and strategies

A survey published by Allianz [10] indicates that many countries have developed their nanotechnology programs up to some levels. Almost all countries covered in this review recognized nanotechnology as an interdisciplinary field involving funding and participation from various organizations/ministries/agencies of government and the private sector. It should be noted that nanotechnology can be carried out independently by federal governments, state/regional governments, agencies, and private players with the proper policy/legal frame work in place; however, best results are usually achieved by networking and collaboration strategies.

Generally, nanotechnology is still at its initial phase of development all over the world. However, advancements made differ from country to country such that nations are grouped on a global scale [11] as

• National activity nations

• Current R/D empowerment nations

• Demonstration of interest nations

Cozzens et al. [12] further classified these countries as very high development, high development, medium development, and low development using the United Nations Human Development Index (UN-HDI). They reported that ‘the last three categories mentioned above combines roughly to be the developing countries’ and of course the LDC. Most African nations belong to the last two categories.

This nanotechnology ranking is simply based on various indicators such as their levels in

• Policy and legal framework

• Funding and investments

• Human resources development

• Industries scenario/economic impact

Nanotechnology is revolutionizing industrial activities in the ‘very high developed and high developed countries’ of the world due to sound policy put in place and huge investment in R/D and infrastructural development. The major players at national activity group include USA, China, Japan, Russia and European countries. Next on current R/D empowerment scale include India, Brazil, Malaysia, Thailand, Singapore, and South Africa among the developing countries, while many countries particularly in Africa are at the lowest level of ‘demonstration of interest stage’ with no budgetary allocations whatsoever.

#### *National activity nations*

Some nations under national activity nations in this our study include USA, Japan, China, UK, Germany, and Russia, among others.

The USA National Nanotechnology Initiative (NNI) launched in 2001 was her first Federal government effort [13]. USA-NNI is under the supervision of National Science and Technology Council, coordinating nanoactivities of more than 25 federal agencies of which 15 have specific nanotechnology budgets. USA has invested about US$15.6 billion for nanotechnology (2001 to 2012) and had her FY2013 budget estimate of about US$1.767 billion [14].

USA has well-established industries investing heavily in nanotechnology such as Hewlett Packard, Motorola, IBM, and Intel in their collaborations with universities. The economic impact is growing speedily with almost over 100 companies in every region of USA focusing on nanoelectronics, semiconductors, pharmaceuticals, and military devices, among others [15]. These achievements have helped create millions of employment and maintain US sustainability and global competitiveness. The activities of organizing and funding nanotechnology initiatives in USA were also carried out by regional, state, and local agencies in their very area of comparative advantages [16].

Japan started her strategic basic research program in nanotechnology in 1995 with various ministries participating headed by the Ministry of Science and Technology. Their launch was based on a 5-year plan, named basic plan, and are relaunched in every 5 years [16]. In the second and third plans, four prioritized research fields were selected in which nanotechnology/materials science is one of the fields. In 2011, about 300 public and private institutions and over 1,200 researchers were involved in nanotechnology activities [17]. Japan is focusing on production of nanomaterial electronics and nanodevices and nanobiomaterials. Japan fashioned their funding into bottom-top category with about 263.3 billion Japanese yen (€2.37 billion) spent in 2011 and top-down research with a budget of about 51.049 million Japanese yen (€477.1 million) [14,17].

China's national nanotechnology programs have existed since 1990 [17], and China appears to be leading the world in the number of nanotechnology companies [16]. The major products of China's nanotechnology are nanomaterials such as nanometal oxides, nanometal powders, and nanocompound powders. Bai [18] reported that ‘China in 2011 had a budget estimate of about €1.8 billion and has instituted her 12^th^ five year plan (2011–2015) rated the most holistic plan anywhere in the world.’ This plan is a target of practical shift from basic research to applied research - mobilizing over 1,000 companies of which a greater percentage of them are domestic SMEs. China like USA included state level participation such as Suzhou Industrial Park and Jiangsu, Shanghai with a total budget estimate of about one billion euros [19].

Irrespective of the great economic challenges facing Europe, seven of the EU countries are actively engaged in nanotechnology activities at their national levels. They include, among others, Germany, France, UK, Spain, Italy, Sweden, Netherlands, and Finland. In Germany for instance, nanotechnology funding stood at about 500 million euros per year with over 750 companies and over 1,000 researchers and 50,000 jobs already created focusing on carbon nanofuel, nanomaterials, and textile, with their industrial partners such as Bayer, EADS, BASF, VARITA, and Siemens [14].

Similarly, France has a budget of about 400 million euros per year with about 130 companies and over 700 nanoresearchers in nanobiotechnology. Again, UK invests about 250 million euros per year with about 200 nanotechnology companies focusing on nanobiotechnology, nanomedicine, nanoenergy, and nanomaterials [14].

Other countries in Europe have their investments at about 100 million euros per year and with well-tailored targets to achieve their interest and maintain global competitiveness and sustainability.

Observatory NANO [14] reported that the Russian government has since 2006 launched their nanotechnology activities with block funding from various government agencies with Federal Agency for Science and Innovation (ROSNAUKA) as the implementing body. They have two main bodies charged with overall activities of nanotechnology: the Russian Corporation of Nanotechnologies - as an agency responsible for commercialization of nanoproducts and innovations targeting to create many nanotechnology industries by 2015 [20]. Another agency is the National Nanotechnology Network - a body charged with responsibility of coordinating activities of over 480 R&D institutions and about 1,700 researchers. The focus of Russia which is on using cluster manufacturing approach is to produce nanomaterials, nanomedicine, nanophotonics, and nanoelectronics for ICT.

#### *Current research and development empowerment nations*

Discussion on the implications of nanotechnology is going on well among developing countries. Many see nanotechnology as an opportunity for further exploitation of the developing countries [21], whilst others see it as an opportunity to promote sustainability by focusing on the gains [22]. Both opinions may be correct for a nation, depending on what they believe and the steps taken.

Court et al. [23] categorized 10 developing countries as either fourth runners, middle ground, or up-comers, while Cozzens et al. [12] reported that the Brazil, Russia, India, and China (BRIC) countries dominate nanotechnology publications in the developing countries. They further reported that there is very little activity outside the BRIC and that ‘The nanotechnology game appears to be largely limited to the affluent countries and the BRIC.’ Clearly, advancements in nanotechnology made in China and Russia is enormous that they are no longer in the same categories with other developing countries hence their inclusion in this study as national activity nations.

There are also a few other developing countries that have joined the BRIC in the fourth runners' category, because they have caught the vision of upcoming nanotechnology industrial revolution, and have started their own nanotechnology initiatives through proper policy framework, robust budgetary plan, network linkages, and human capital development for successful national development in line with the effort of Asian and Pacific Centre for Transfer of Technology-United Nations Economic and Social Commission for Asia and the Pacific (APCTT‒UNESCAP) to facilitate regional collaborations in nanotechnology innovation and industrial application [24]. These countries include South Africa, Malaysia, Singapore, Sri Lanka, Taiwan, and Thailand. Many other countries are at various stages of unknown level either at current R/D empowerment or demonstration of interest stage [11,25,26].

Brazil first launched her nanotechnology program in 2005 with a budget of about US$31 million with 10 research networks involving about 300 PhD researchers [27]. Their focus has been on nanoparticles, nanophotonics, nanobiotechnology, CNTs, nanocosmetics, and simulation and modeling of nanostructures. Brazil has a strong collaboration link in her plan 2007 to 2013 with European Union, South Africa, and India, which has strengthened their nanotechnology capabilities.

TERI [28] reported that active Nanoscience and Technology Initiative (NSTI) started in India when its government launched her 5-year plan 2007 to 2012 with a budget estimate of US$254 million (approximately Re1,000 crore). The plan was aimed at developing centers of excellence (COEs) targeting laboratories, infrastructure, and human resource development. They have strong collaboration with foreign stakeholders. Many of her states are participating actively in nanotechnology programs such as Karnataka, Trivandrum and Tamilnadu engaging in biotechnology and health-related activities, respectively. The India Department of Science and Technology (DST) is the agency responsible for both basic and applied research in nanotechnology, with their areas of focus include nanotubes, nanowire, DNA chips, and nanostructured alloys/systems, among others.

Molapisi [29] reported that South Africa is at the forefront and had strategically started her nanotechnology activities with a budget of US$2.7 million in 2005 and has spent a total sum of about US$77.5 million (2005 to 2012). South Africa nanotechnology is powered by her DST focusing on human capital development through students on researcher support program, establishment of nanoscience centers, equipment acquisition program, and establishment of nanotechnology platform and two nanotechnology innovation centers that will encourage patent and prototype products [26]. South Africa has a strong collaboration with foreign partners especially Brazil and India. Today, South Africa has gone into applied research stage focusing on nanocatalyst, nanofilters, nanowires, nanotubes, and quantum dots [28].

Malaysia started her nanotechnology campaign in 2001 and categorized it as a strategic plan under her IRPA (8MP) 2001 to 2005. A more robust plan was made for a 15-year period from 2005 to 2020 with more than 150 local researchers focusing on nanotechnology for advance materials and biotechnology to encourage the development of new companies and new products [30].

Wiwut [31] reported that in Thailand, the National Nanotechnology Center (NANOTEC) was approved in 2003 with National Science and Technology Development Agency under Ministry of Science and Technology supervising with a mandate to promote industrial clusters in nanotechnology through human resource capitals and robust infrastructural development. Thailand's program receives approximately US$2 million per year [32] in which the success recorded so far is by collaborative networks in research and funding by various government agencies through their various COEs in nanotechnology. Their major focus is on nanobiotechnology, nanoelectronics, nanomaterials, and nanocomposites.

Similarly, Singapore has an elaborate nanotechnology capabilities utilizing nanomaterials, nanodevices in microelectronics/MEMS fabrications, clean energy, and medical technology, among others, in so many well-established nano-SMEs involving technology/manufacture and sales/marketing under government funding and collaborative arrangements [33].

A greater lesson and of special interest to Africans should be that of Sri Lanka, a country of about 20 million people and primarily of an agricultural-based developing economy but with visional leaders who, through its Ministry of Science and Technology and National Science Foundation (NSF), recognize the importance of nanotechnology in the oncoming industrial revolution. Nanoglobe [24] reported that ‘Sri Lanka, though with limited infrastructure built for R&D and limited funding from the government so far, shows its commitment in developing nanotechnology with a unique private public partnership and passionate scientists. Sri Lanka NSF launched its Nanotechnology Initiative in 2007 and set up the Sri Lanka Institute of Nanotechnology (SLINTEC) as a private company with LKR 420 million (about US$3.7 million) in 2008 with a unique public private‒partnership (PPP) structure where 50% of institute funding comes from 5 private companies including Hayleys, MAS Holdings, Brandix, Loadstar and Dialog.’ This Sri Lanka approach is a typical lesson for Africa and LDC governments to learn from.

Nanoglobe [24] and Sarka et al. [34] reported that Iran had its National Nanotechnology Initiative launched in 2005 for a 10-year period up to 2015 with broad mark achievements. Meanwhile, half of its nanotechnology budget is funded by the private sector, with her scientists and industries actively engaging in international cooperation activities. It has an established education program to train MSc and PhD students in about 50 universities and research institutes. Its R&D priorities are energy, health, water and environment, nanomaterials, and construction. Iran is heading the Asian Nano Forum (ANF) Energy and Water Working Group.

Su et al. [35] reported that the Taiwan National Science and Technology Program for Nanoscience and Nanotechnology was initiated in 2002 and aims to achieve academic excellence in basic research and accelerate nanotechnology commercialization. The project has four segments - academic research excellence, industrial techniques, talent search, and establishment of core facilities. Her target is at consumer goods, metal oxides and machines, chemicals, electronic and information technology, energy, and biotechnology. Taiwan has well-equipped centers such as the Nanotechnology Research Center established by Industrial Technology Research Institute (ITRI), the Nanoscience Laboratory of Academia Sinica, and National Nanocomponent Laboratory established by the National Science Council. Through these centers, she coordinates multidisciplinary and multiagency research teams in academic research and promotes industrialization of nanotechnology with about 175 companies participating.

APCTT-UNESCAP [36] reported that serious nanotechnology is ongoing in the Philippines. They have developed a road map towards successful nanoscience and nanotechnology by way of proper policy formulations and definite goals set as targets. Again, her governments have put in place incentives that will lure their scientists abroad to return and help in their science and technology development.

#### *Demonstration of interest nations - African nations and LDC*

Many developing countries are at various stages of unknown level either at current R/D empowerment or demonstration of interest stage [11,25,26]. Apart from South Africa, most countries in Africa are at the demonstration of interest stage in their nanotechnology development effort. Many have not even indicated interest, while those that indicated are not having enough drive to push for success [37]. These African nations are only at the level of individual research and incidental funding [38]. Recently, on August 7, 2012 in Abuja, Nigeria, the Federal Ministry of Environment signed a joint agreement to promote training and capacity building for the development of a nanosafety pilot project in Nigeria with financial support from the government of Switzerland - the overall aim was to create awareness [38]. Zainab [39] reported that ‘nanotechnology is a new field in Nigeria, and systematic efforts are being made by the academia, research institutes and government to create awareness and interest in nanotechnology development.’

Nigeria is one of the up-comer nations with nothing in place indicating nanotechnology activities and the big question is: When will such rich nation like Nigeria key into this technological revolution and practically start their own nanotechnology programs? This is because most of these countries are for too long standing at this demonstration of interest stage not necessarily because of fund scarcity but probably because of political issues that blind them against realities of life. This is true when some of them are by far richer than Sri Lanka with GDP per capita of about US$2,000 [24] yet shows high commitment in developing nanotechnology with a unique private-public partnership and dedicated scientists. We think the problem is basically because there is no well-developed materials science research curriculum and infrastructural platform in these countries upon which such sensitive research can stand. Most universities/research institutes in Nigeria and indeed Africa lack the basic materials characterization equipment at macro- and microlevels and therefore have no understanding of this global trend in nanoscale approach of materials synthesis and characterization. In Nigeria, the highest form of nanotechnology activity is individuals or groups conducting research on nanoparticle synthesis and application in polymers and composite materials [39].

Nanoglobe [24] and APCTT-UNESCAP [36] also reported that Bagladesh and Nepal have not launched nanotechnology initiatives due to their limited infrastructure for R&D, lack of trained human resources, and limited international collaboration. In Nepal, there are research groups conducting research on nanoparticle synthesis and application in polymers and composite materials, while in Bangladesh, the Materials Science Division of Atomic Energy Centre at Dhaka is carrying out some research work in the field of nanotechnology covering some selected areas.

It is clear from this study that most African nations and LDC share a similar story where basic research laboratory facilities is lacking from university to university and from one research institute to another, yet some of them earn huge revenues from their natural resources. This state of no action classifies Nigeria and other countries alike as nanotechnology-dormant nations since there is nothing going on as relating to nanotechnology except conferences and selective individual/group research efforts.

### Opportunities and challenges of nanotechnology for Africa and LDC

The evolution of nanotechnology is at its early stage globally, and Cozzens et al. [12] reported that ‘applying nanotechnology to meeting the Millennium Development Goals for 2015 remains as far away as it was in 2005, even though the target date is much closer. This is because nanotechnology activities are very much dominated by laboratories in the global North and the BRICs countries without any activity in some developing countries.’ This is a great global challenge and yet an opportunity for advancements. Yes, it is an opportunity through which developing countries can become part of the industrial shaping and through such participation strengthen their technological capacity, capabilities, and sustainability. Some developing countries that have come to this knowledge are investing heavily in it, such as India, Brazil, China, Thailand, and South Africa, among others.

Maclurcan [40] rightly reported that the manner and way in which some developing countries are going about their nanotechnology engagement is believed to be as largely given and as passive actors which, if not attended to, will turn them into perpetual nanotechnology importers thereby increasing their economic and technological dependence on the developed countries worse than today's experience. He suggested that an early developing country engagement with nanotechnology innovation could reduce the possibility of these countries being net importers of the technology.

The challenges of a country engagement in nanotechnology, however, are enormous and some of them seem insurmountable simply because of the political issues dominant in most developing countries, but the truth is that nanotechnology is here with us and will soon form part of our everyday product usage. Some of the challenges of nanotechnology development in third world nations as reported by Babajide [25] include but not limited to the following:

• Lack of proper legislation/regulatory framework and the relevant political drive

• Lower government spending on research and development (R&D)

• Lack of infrastructure and human capacity

• Lack of proper education relating to curriculum development matters

• Lack of private enterprise participation in research and development

• Lack of proper collaboration and network programs among agencies

• Research institutes and industries that will translate basic research into applied research and end products

• Poor industrialization status of the third world countries

• Inadequate foreign linkage particularly with donor agencies in nanotechnology

• Fear of health, environmental, and safety risks associated with nanotechnology

### Lessons for Africa and LDC - the nanotechnology way forward

Various lessons can be learnt from this discussion on nanotechnology initiatives for African nations and other LDC, which they can adopt as practical steps to establish a robust nanotechnology program in their country. These lessons include but not limited to the following:

1. A ministry of nanotechnology or a department of nanotechnology should be created under the ministry of science and technology to focus on human capital development through students on researcher support program as well oversee the general activities of nanotechnology in the nation.

2. A strong collaboration link between African nations and nations like South Africa, India, and European Union which has strong nanotechnology capabilities should be established in order to help guide them on various areas of nanotechnology activities including funding.

3. The nation's policy formulations and definite goals should favor nanoscience and nanotechnology such that inclusion of nanotechnology budget in relevant ministry of government is guaranteed.

4. African nations and LDC can only make a headway in the activities of nanotechnology by making enormous budgetary allocations to research and development of nanotechnology and indeed launch the NNI formally like other nations that are already advanced in nanotechnology programs.

5. A wide campaign through seminars/symposiums should be carried out through universities/governmental agencies so as to recognize the importance of nanotechnology in the oncoming industrial revolution.

6. Private companies should be encouraged to partner with the public sector in funding nanotechnology programs with a view to develop nanotechnology and improve the nation's economy.

7. Short- and long-term plans on nanotechnology should be set in motion to promote the development of new companies, new products, and advance materials.

8. African nations and other LDC should emulate a nation like Iran who has established education program to train MSc and PhD students in the universities and research institutes in the area of nanotechnology having its priorities on energy, health, water and environment, nanomaterials, construction, and other priority areas.

9. Centers of excellence in nanotechnology research and development should be established with state-of-art facilities for nanotechnology in African universities and research institutes. In these centers, specialized trainings can be organized for personnel as to fast improve on human resource requirements.

10.  States and viable local governments should be encouraged as much as possible to start their own independent nanotechnology initiatives/programs in their various areas of interest. In other words, all government levels: federal, state, and local should be mobilized to enter into linkage/collaboration with developed countries in terms of training and development of human resources such as sponsoring at least three PhD students in nanoscience and technology for training/fellowship abroad on annual basis for the next 10 years.

11.  The government of African nations should encourage established industries within the country (expatriate/indigenous companies) to explore the area of nanotechnology in their future investments. These industries should work in collaborations with universities in these areas of research.

12.  Government and researchers can establish nanoscience centers or float nanotechnology companies that will promote a specific nanoproduct to ensure technological growth and enhance the economy of the nation as well. This will promote employment/job activities in nanotechnology (especially in the area of research and development).

13.  Research grants should also be made available to Masters/PhD students willing to work in this area.

14.  Researchers in research institutes should also be motivated by giving them reasonable incentive in the form of research grants and all forms of moral support.

15.  Government and researchers should focus on our available natural resources: how they can be harnessed/maximized using nanotechnology.

## Conclusions

Nanotechnology is the material transformation, advancement, and development of our time. Many nations of the world including some developing countries have since launched their nanotechnology programs and are at various levels of success. African nations and indeed other developing nations at the expression of interest stage can also embrace the challenges with vigor and determination to make it by establishing a fortified nanoscience/nanotechnology program in their country through proper curriculum development, timely legislation, and budgetary funding/investment and collaborations in partnership with the private sector and donor nations/agencies. The long-term economic benefits will surely increase the country's sustainability and global competitiveness.

Perhaps the health issues associated with nanoproducts may be one of the impending factors against developing countries' participation in nanotechnology. It is therefore necessary that a more independent research on the overall health risk associated with nanoproducts be made very transparent and available to all concerned. In light of this, various governments of the world should consistently encourage nanotechnology health risk research as it may concern them with adequate funding to achieve objective results within an objective and proper legislative framework. LDC and African nations in particular should urgently review her tertiary education programs to give the much desired attention to nanomaterials testing, synthesis, and characterization using state-of-art equipment; otherwise they may be promoting the much talked about ‘nano divide’ of which they will suffer more as consuming nations. The time to act is now.

Finally, African nations and LDC should endeavor to utilize the window of cooperation and collaboration now available with developed countries such as USA, European Commission, China, and Japan to enable them to access assistance. This assistance may be sorted through proper training of her human capacity and funding/donation of equipment from these developed nations and multinational agencies which is specifically meant for nations at the demonstration of interest stage. This very window is wide open now but will not remain so for a long time. African nations and other LDC should not allow such opportunity to waste away. The earlier they make advances to the realities of nanotechnology, the better their nations will be.

It is only when these steps are taken that African nations and other LDC can apply nanotechnology innovatively to improve the quality of life of her citizens, thus enabling local industries and businesses to strive for sustainability and competitiveness in today's global business setting. The emphasis is on PPP and networking through responsible development and regulatory framework by all government ministries, agencies, and stakeholders.

We are calling on the laboratories of the developed countries and the BRIC to urgently take up these challenges of the developing countries if our dream of global integration is to be real. The time for this assistance is now.

## Competing interests

The authors declare that they have no competing interest.

## Authors' contributions

ICE carried out the extensive survey via internet and drafted the manuscript, POO formulated the topic and participated in formatting and proof reading of the manuscript. ADO helped in drafting the section ‘Lesson for Africa and LDC.’ He also proof read the manuscript for grammatical/typographical errors when the need arises. All authors read and approved the final manuscript.
